# Development and Usability Evaluation of an E-Learning Tool for Blended Learning in Pediatric Endocrinology: Formative Pilot Study

**DOI:** 10.2196/89064

**Published:** 2026-07-21

**Authors:** Farah Hrasnica, Sophie Pitteloud, Jessica Jacot, Maria Christina Antoniou, Inge-Lore Ruiz-Arana, Thérèse Bouthors, Kanetee Busiah, Sophie Stoppa-Vaucher, Michael Hauschild

**Affiliations:** 1 Faculty of Biology and Medicine, University of Lausanne, Lausanne, Vaud, Switzerland; 2 Department Women-Mother-Child, Pediatric Endocrinology, Diabetology and Obesity Unit, University Hospital of Lausanne, Lausanne, Vaud, 1011, Switzerland, 41 21 314 11 11; 3 Department of Endocrinology-Growth and Development, Panagiotis & Aglaia Kyriakou Children's Hospital, Athens, Attica, Greece; 4 Pediatric endocrinology and diabetology unit, Children’s Hospital, Luzerner Kantonsspital, Lucerne, Lucerne, Switzerland; 5 Department of pediatrics, Réseau hospitalier Neuchâtelois, Neuchâtel, Switzerland

**Keywords:** medical education, e-learning, blended learning, andragogy, implementation

## Abstract

**Background:**

Residents in pediatric endocrinology subspecialty units encounter diverse educational scenarios spanning theory, skills, and attitudes; yet, brief residencies frequently limit their exposure to certain clinical cases. Research in medical education demonstrates that e-learning can address such challenges efficiently. We implemented a blended learning model grounded in the Kolb learning cycle that uses structured, case-based e-learning.

**Objective:**

We aimed to evaluate the utility and usability of blended learning using a novel e-learning tool.

**Methods:**

We used a problem-solving approach and used the physical separation of case-based e-learning (interactive, patient scenario–based online modules) and theoretical content delivery as the educational model for residents in a pediatric endocrinology and diabetology unit. Residents worked asynchronously (on their own time, not simultaneously with others) on clinical scenarios and completed formative assessments (practice tests designed to provide feedback for learning rather than grades) with immediate feedback using a flipped classroom teaching method, in which students review material before group instruction. In addition, all cases could be discussed with specialists during face-to-face learning opportunities through a blended learning approach that combines online and in-person elements. We evaluated Kirkpatrick level 1 (reaction, how participants respond to training) and level 2 (learning, measured as an increase in knowledge or capability) outcomes using the postgraduate Medical E-learning Evaluation Survey (MEES) and the User Experience Questionnaire (UEQ), which assesses users’ perceptions of e-learning platforms.

**Results:**

Questionnaires from 12 pediatric residents and 1 questionnaire from a fourth-year medical student were evaluated. The main strengths identified were the tool’s support for applying content to daily clinical work (12/13, 92% users), provision of timely summaries (n=9, 69% users), access to reliable information sources (n=9, 69% users), and immediate feedback on responses (n=8, 62% users). Key weaknesses included device compatibility for e-learning (n=5, 38% users), limited content personalization (n=4, 31% users), and a lack of a navigation aid (n=4, 31% users). No significant functional issues were reported. The UEQ evaluation showed that dependability received the lowest rating, while attractiveness and stimulation received the highest rating.

**Conclusions:**

Our e-learning proposal provides a practical way to apply theoretical knowledge through interactive clinical cases. Evaluations show that users are highly motivated to engage with e-learning, highlighting our tool’s adaptability and effectiveness for postgraduate medical education in pediatric endocrinology. Identifying strengths and weaknesses will guide future improvements. Evaluating various aspects of e-learning remains crucial, as these aspects can affect learning outcomes. However, more longitudinal evaluations of e-learning are necessary to achieve a comprehensive understanding of its effectiveness.

## Introduction

Learning involves acquiring knowledge, skills, and attitudes through a dynamic, multifaceted process influenced by motivation, context, and community. These factors shape personal learning styles and behavioral, psychological, and cognitive steps [1].

Theoretical knowledge is a foundational element of Miller’s pyramid of clinical competency [2]. However, it is the practical application of this knowledge and the development of clinical skills that truly lead to clinical competency. This hands-on experience is essential, and its importance cannot be understated in our rapidly evolving work context and with the emergence of AI interference.

Postgraduate medical education, a phase during which long-term, evidence-based practical knowledge, clinical skills, and professional attitudes are acquired, is a significant part of a medical professional’s journey. In this context, Kolb’s experiential learning model [3], which describes an integrated process consisting of 4 stages (concrete experience, reflective observation, abstract conceptualization, and active experimentation), is particularly relevant. Its alignment with the characteristics of adult learning makes it highly applicable to medical education.

Not all clinical situations can be experienced during short-term residencies (3-6 months) in specialties such as pediatric endocrinology. Problem-based and case-based formats effectively support applied learning and knowledge acquisition in medical education [4]. This aligns with the principle of storytelling in higher education, where structured narratives help medical residents connect theory and practice and reflect on their reasoning and decision-making processes [5]. E-learning offers a complementary opportunity for asynchronous blended learning [6-8].

*E-learning* can be defined as “an approach to teaching and learning, representing all or part of the educational model applied, that is based on the use of electronic media and devices as tools for improving access to training, communication, and interaction that facilitates the adoption of new knowledge, skills, and/or behavior and attitude” [9]. Internet-based medical education has quickly become an essential tool [6,10-12]. The instructional design of e-learning models can be classified into four categories [13]: (1) serious gaming, which uses game-like simulations to teach medical concepts; (2) virtual reality, which provides immersive experiences for medical training; (3) simulation, which replicates real-world medical scenarios for practice; and (4) e-learning aimed at theoretical knowledge, which focuses on delivering theoretical medical knowledge. Additionally, the use of animated pedagogical agents (APAs) is described as an effective and useful component of instructional design [14,15].

There is also a need to consider the learning environments in which learners evolve, as these environments influence learning effectiveness and motivation. The expectancy-value model states that learners’ engagement and persistence in an activity depend on their confidence in succeeding and the value they attribute to a task, weighed against perceived costs, such as effort and stress [16]. The level of cognitive engagement in an activity can be described using the Interactive, Constructive, Active, and Passive (ICAP) framework, which categorizes engagement from passive to interactive and links higher engagement to deeper learning [17]. AI is increasingly used in clinical settings as a “knowledge finder.” However, using AI for passive research cannot replace engaged, active learning models such as e-learning, and its contribution to the learning cycle remains unclear [18,19].

Evaluating medical e-learning is a complex, heterogeneous process. Most studies focus on Kirkpatrick levels for evaluating learning outcomes [13]. The 4 levels are reaction (level 1), learning (level 2), behavior (level 3), and results (level 4) [20]. In their systematic review, de Leeuw et al [13] emphasize the importance and necessity of including instructional design in the evaluation process. This complexity underscores the need for a comprehensive assessment of medical e-learning.

For our pilot study, we aimed to develop and evaluate the usability of an e-learning tool in a blended learning environment during a short-term clinical internship in a specialty medical environment. It includes structured case-based e-learning courses based on the European Training Curriculum in Pediatric Endocrinology and Diabetes of the European Society for Paediatric Endocrinology (ESPE) [21], complemented by a theoretical knowledge platform, and face-to-face learning approaches during clinical work. Our pilot study may serve as a basis for the development of future e-learning tools. It also tackles the issue of problem-oriented learning during short-term residencies in clinical subspecialties for medical residents.

Our hypothesis is that the e-learning tool will be perceived by residents as motivating, usable, and educationally effective. Positive user experience (eg, usability, efficiency, and stimulation) will be positively associated with motivation and negatively associated with perceived barriers.

## Methods

### Overview

A freely accessible, dedicated knowledge-sharing website, including a link to the e-learning website, was designed by the Lausanne University Hospital audiovisual communication and creation service. We used the user-friendly Moodle (Moodle Pty Ltd) learning management system at Lausanne University Hospital to build and implement the pediatric endocrinology e-learning platform [22]. This e-learning platform required log-in and personal registration. The 2021 Pediatric Endocrinology syllabus [21], published by the ESPE, served as the foundation for the chapter structure, the selection of topics addressed through the e-learning platform, and the formulation of specific learning objectives. Macro objectives encompass broader aspects, including general knowledge of the subject, identification of red flags, and patient management, among other competencies. Micro-objectives are specific to individual clinical cases and correspond to objectives that can be achieved by completing the interactive activities on the e-learning platform. Regarding the clinical cases, we used data from real patients, for whom consent and/or parental consent for data collection had been obtained previously at Lausanne University Hospital.

We used the open source HTML5 package (H5P; D2L Inc) [23] module integrated into the Moodle platform to transform clinical situations into structured interactive content, including comprehensive learning objectives, reactivation of prior knowledge, current history, prior history, familial history, physical examination, additional examinations, therapeutic interventions, and key messages with links to an openly accessible content-sharing website named “Pedlaus” [24]. In addition, the e-learning platform can be accessed directly from the Pedlaus website. The instructional design of our e-learning tool was guided by evidence-based principles of multimedia learning, incorporating signaling of key elements, integration of text and visuals, learner control, and immediate formative feedback [25]. It also incorporates problem-based learning through interactive clinical cases. All clinical cases and content were meticulously written and rigorously validated by the medical staff of the Pediatric Endocrinology, Diabetes, and Obesity Unit at Lausanne University Hospital. Additionally, we created a user guide to help learners navigate the platform.

We evaluated our e-learning tool using the postgraduate Medical E-learning Evaluation Survey (MEES) developed by de Leeuw et al [26] and the User Experience Questionnaire (UEQ) developed by Schrepp et al [27]. These instruments enabled us to assess Kirkpatrick level 1 and part of level 2. As we completed a formative assessment of our e-learning tool, we did not evaluate the achievement of the learning objectives.

The MEES aims to determine the usefulness, understandability, and added value of postgraduate medical e-learning modules such as ours. The questionnaire assesses 5 domains using a single 10-point Likert scale: motivators, barriers, learning enhancers, learning discouragers, and real-world translators. As each construct was measured with 1 item, internal consistency reliability statistics such as Cronbach α were not applicable.

The strengths of e-learning are assessed through the motivators, learning enhancers, and real-world translators domains. The weaknesses are assessed through the barriers and learning discouragers domains. Users could also add their comments to each assessed domain [26].

The UEQ can be used to evaluate the user experiences with interactive online products. The questionnaire comprises 26 items that quantitatively evaluate 6 categories: attractiveness, perspicuity, efficiency, dependability, stimulation, and novelty. The results are represented on a 7-point Likert scale, ranging from “–3” to “+3” and including a moderate or neutral midpoint. Values between –0.8 and 0.8 represent a neutral evaluation. If values are >0.8, it is a positive evaluation; if they are <−0.8, it is a negative evaluation. We then proceeded to a descriptive analysis of the results using the calculations recommended by the authors, including Cronbach α [27]. The latter serves as a measure of scale consistency and reliability. A scale is generally considered acceptable if Cronbach α is ≥0.7. Finally, the results were compared with the UEQ benchmark, which is updated annually and includes data from 452 UEQ product evaluations (N=20,190 participants) [28].

We evaluated our e-learning tool in a blended learning environment, combining asynchronous self-directed e-learning with optional face-to-face discussions with senior specialists.

Our pilot study followed the Guidelines and Checklist for the Reporting on Digital Health Implementations (iCHECK-DH) [29] (Checklist 1).

### Ethical Considerations

This study was conducted in accordance with the Declaration of Helsinki. According to institutional regulations of the Lausanne University Hospital Ethics Committee, formal ethics committee approval was not required for this type of educational research. The study involved an educational evaluation of an e-learning tool and did not include direct patient involvement. Medical residents participated voluntarily, and informed consent was obtained. For clinical case creation, written informed consent for the use of anonymized medical imaging and laboratory data for medical education was obtained from patients and/or legally authorized representatives.

## Results

### Overview

A total of 27 clinical cases, based on real patients, were developed based on the macro objectives of each chapter, including relevant issues of general knowledge of the subject (physiopathology and clinical implications), as well as specific clinical, laboratory, and imaging learning points. A total of 13 participants (n=12, 92% medical residents and n=1, 8% fourth-year medical student) tested the e-learning tool and completed the 2 questionnaires. Multimedia Appendix 1 shows several screenshots of the e-learning tool, offering a general overview.

### User Experience Questionnaire

**Figure 1.**
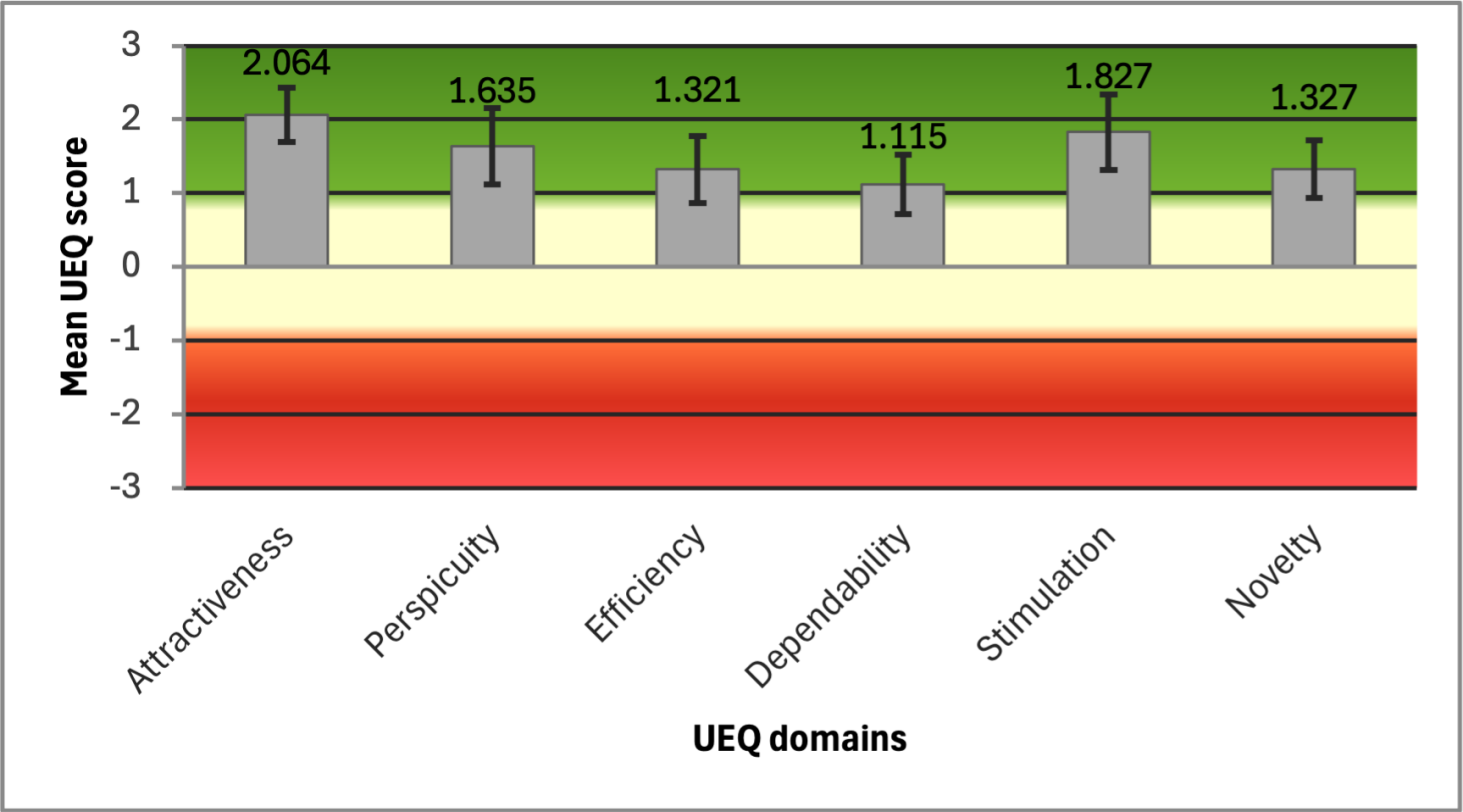
Mean scores and variance for each evaluated category of the User Experience Questionnaire reported by the 13 participants (12 medical residents and 1 medical student) in the formative evaluation of the pediatric endocrinology e-learning tool. Values between –0.8 and 0.8 indicate a neutral evaluation, values >0.8 indicate a positive evaluation, and values <–0.8 indicate a negative evaluation.

All 6 UEQ categories were positively evaluated. Attractiveness (mean 2.064, SD 0.672) and stimulation (mean 1.827, SD 0.949) were the highest-rated categories, whereas dependability was the lowest-rated category (mean 1.115, SD 0.740; Figure 1).

**Figure 2.**
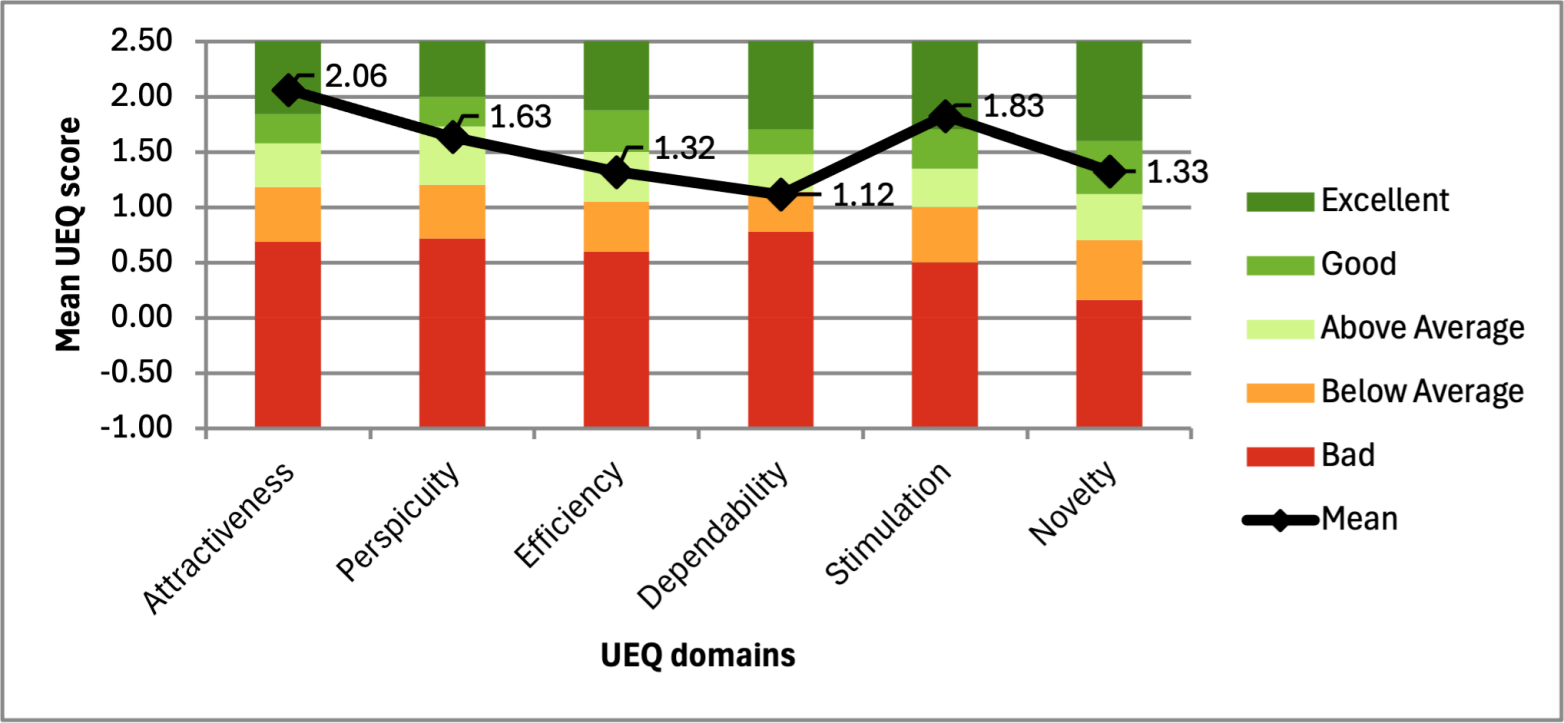
Benchmark comparison of the User Experience Questionnaire results for our e-learning module. “Excellent” indicates that the result for the evaluated product is in the range of the 10% best results, “good” indicates that 10% of the results in the benchmark dataset are better than the result for the evaluated product and 75% of the results are worse, “above average” indicates that 25% of the results in the benchmark dataset are better and 50% of the results are worse, “below average” indicates that 50% of the results in the benchmark dataset are better and 25% of the results are worse, and “bad” indicates that the result for the evaluated product is in the range of the 25% worst results.

Among the 26 items, 23 received a positive evaluation, and 3 received a neutral one: creative or dull (mean 0.8, SD 1.6), unpredictable or predictable (mean 0.5, SD 0.9), and fast or slow (mean 0.5, SD 1.6). Figure 2 presents the benchmark comparison. Table 1 shows the CIs for each category. Table 2 presents the reliability coefficients for each scale’s category (Cronbach α). The scales for attractiveness, stimulation, perspicuity, and efficiency demonstrated acceptable reliability, with Cronbach α >0.7. In contrast, dependability and novelty showed lower reliability, with Cronbach α <0.7.

**Table 1.** Mean User Experience Questionnaire domain scores, SDs, and CIs (*P*=.05 per scale) among the 13 participants (12 medical residents and 1 medical student) in the formative evaluation of the pediatric endocrinology e-learning tool.

Domains	Values, mean (SD; 95% CI)	95% CI margin
Attractiveness	2.064 (0.672; 1.699-2.429)	0.365
Perspicuity	1.635 (0.950; 1.118-2.151)	0.516
Efficiency	1.321 (0.837; 0.866-1.775)	0.455
Dependability	1.115 (0.740; 0.713-1.518)	0.402
Stimulation	1.827 (0.949; 1.311-2.343)	0.516
Novelty	1.327 (0.717; 0.937-1.717)	0.390

**Table 2.** Cronbach α coefficients for internal consistency of multi-item scales for the User Experience Questionnaire (UEQ) among 13 participants (12 medical residents and 1 medical student) in the formative evaluation of the pediatric endocrinology e-learning tool^a^.

UEQ domains	Cronbach α (5% CI)
Attractiveness	0.85 (0.62 to 0.94)
Perspicuity	0.74 (0.32 to 0.90)
Efficiency	0.74 (0.33 to 0.90)
Dependability	0.40^b^ (−0.58 to 0.77)
Stimulation	0.85 (0.62 to 0.94)
Novelty	0.51 (−0.27 to 0.82)

aα values ≥0.7 are generally considered acceptable.

b The lower Cronbach α for dependability may be due to the small sample size and the multidimensional nature of the construct.

### Postgraduate MEES

The average score for each evaluated domain is represented in Multimedia Appendix 2. In addition to rating each domain, users selected specific items corresponding to motivators, barriers, learning enhancers, and other categories. The most frequently selected items and comments are illustrated in Textbox 1, along with items that were not chosen. Motivation, learning enhancers, and real-world translation were all highly rated (Multimedia Appendix 2), highlighting several strengths of the e-learning tool. Motivation has a mean evaluation of 8 out of 10, learning enhancers 8.08 out of 10, and real-world translation 8 out of 10. Out of 11 motivators, 4 (36%) were selected by more than half of the users (Textbox 1; motivators 1, 5, 8, and 9). Similarly, 4 out of the 8 learning enhancers (Textbox 1; enhancers 1, 5, 6, and 7) and 3 out of the 4 real-world translators (Textbox 1; translators 1, 2, and 3) were selected by more than half of the users. Among the categories assessing weaknesses, barriers received a slightly more negative overall evaluation, with a score of 3.23 out of 10, than 2.69 out of 10 for learning limitations (Multimedia Appendix 2). However, none of the barriers or learning discouragers were selected by more than half of the users (Textbox 1). The medical student’s responses were aligned with those of the medical residents in the real-world translation domain.

Textbox 1. **Medical E-learning Evaluation Survey items selected by at least 50% (n=7) or none of the 13 participants (12 medical residents and 1 medical student) in the formative evaluation of the pediatric endocrinology e-learning tool.**

**Motivators**

- Item 1: “I felt this e-learning was important.” (n=7, 54%)
- Item 5: “The e-learning objectives (for each educational section) were clear to me.” (n=7, 54%)
- Item 8: “I felt comfortable with the quality/truthfulness of the content.” (n=7, 54%)
- Item 9: “I was able to do this e-learning unforced.” (n=7, 54%)
- Item 2: “I felt it was my responsibility to do this e-learning.” (n=6, 46%)
- Item 6: “There was a clear overview of all content.” (n=6, 46%)
- Item 10: “I felt taken seriously as an adult learning.” (n=6, 46%)
- Item 4: “I had a good understanding of the general purpose of the e-learning.” (n=5, 39%)
- Item 11: “The e-learning was aimed at my level of experience.” (n=5, 39%)
- Comment 1: “Clarity of the summaries.”

**Barriers**

- Item 1: “I was not able to create my own learning path to my own needs.” (n=4, 31%)
- Item 5: “There was no instrument to help me navigate the e-learning (for example a sitemap).” (n=4, 31%)
- Item 6: “I had worries about the security and safety of the e-learning, regarding my personal information.” (n=4, 31%)
- Item 2: “The e-learning was not easily accessible at my location or with my device.” (n=2, 15%)
- Item 8: “I did not know which devices the e-learning was compatible with and I might have used the wrong one.” (n=2, 15%)
- Item 7: “The e-learning was slow and took too long to load.” (n=0, 0%)
- Item 9: “The e-learning was too long.” (n=0, 0%)
- Comment 2: “Chapter headings with different fonts and sizes make reading/navigation unclear initially.”
- Comment 3: “Variety of materials offered (video, PowerPoints) make it difficult to find one’s way around; too long videos can be discouraging.”

**Learning enhancers**

- Item 5: “The e-learning provided summaries when needed.” (n=9, 69%)
- Item 6: “The e-learning provided feedback on my answers.” (n=8, 62%)
- Item 1: “I could personalize the e-learning (for example by saving and continuing, filling out questionnaires and getting my personal score, etc).” (n=7, 54%)
- Item 7: “There were exercises and/or assignments in the e-learning.” (n=7, 54%)
- Item 2: “I could create my own learning path, and was not forced to follow the directed path (for example by skipping parts or returning to previous sections if needed).” (n=6, 46%)
- Item 8: “I could interact with the content of the e-learning (for example questions, exercises or other interactivities).” (n=6, 46%)
- Comment 4: “The sidebar is useful, but it is difficult to know if it is necessary to follow a particular order or not.”

**Learning discouragers**

- Item 2: “The content was not able to adapt to my device when needed (for example, the e-learning should work on mobile device, but the icons were too small for that).” (n=5, 39%)
- Item 1: “I got stressed or frustrated by the e-learning for any reason.” (n=3, 23%)
- Item 3: “The e-learning design and visuals were too distracting for me.” (n=0, 0%)
- Comment 5: “Is it necessary to follow the order?”
- Comment 6: “Mix of different materials difficult, long videos.”
- Comment 7: “Not always adapted to my level of paramedical training.”

**Real-world translators**

- Item 1: “The e-learning content and examples are translatable to my daily real-world work.” (n=12, 92%)
- Item 3: “The e-learning provided sources for the information which were also accessible after finishing it.” (n=9, 69%)
- Item 2: “The e-learning seems up-to-date and maintained.” (n=8, 62%)
- Item 4: “Besides this questionnaire, the e-learning was evaluated on topics like user experience, effectiveness, usability and/or costs.” (n=0, 0%)
- Comment 8: “Visible dates and references lack in order to know if it’s updated.”

## Discussion

### Principal Findings

**Figure 3.**
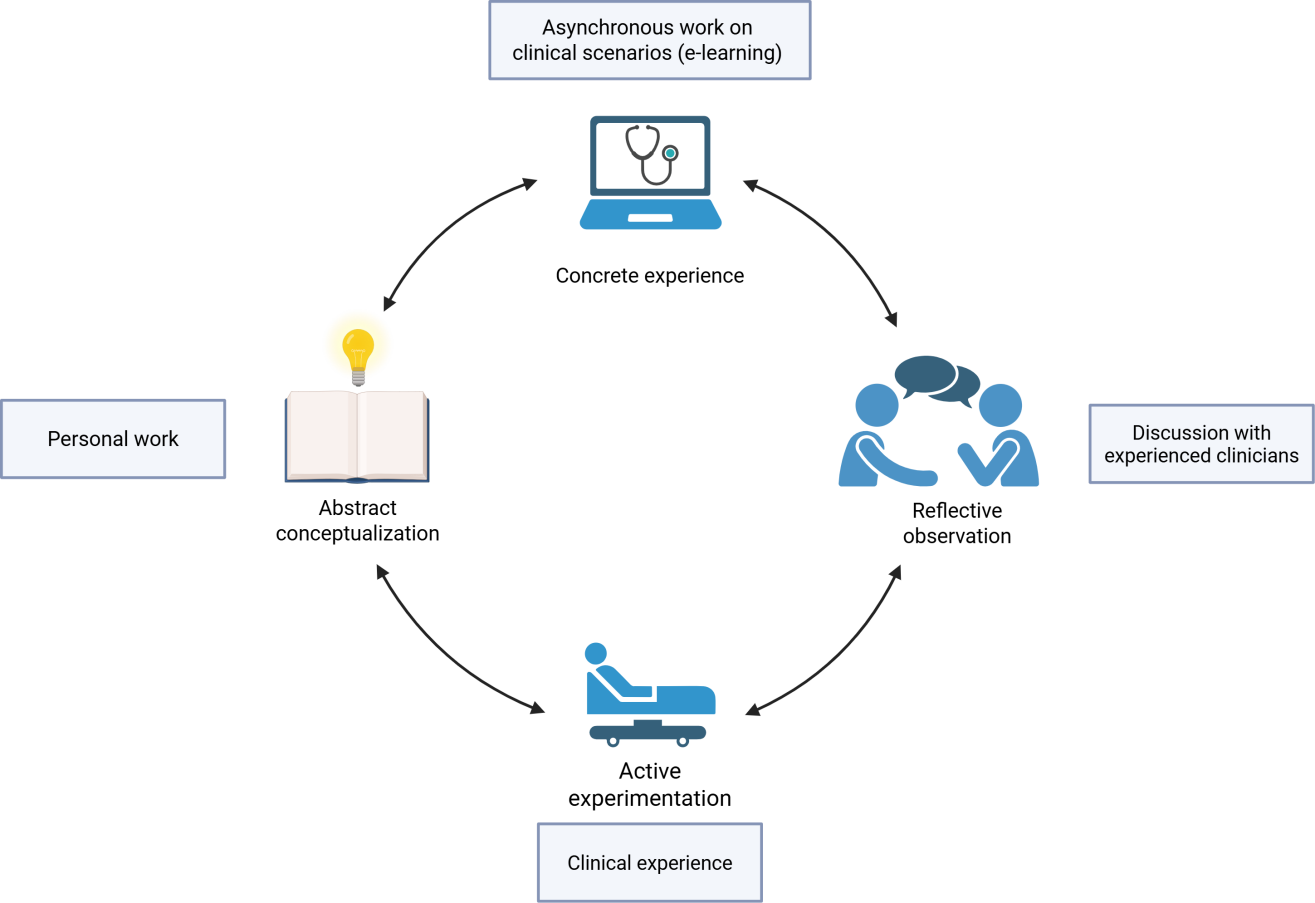
Mapping of Kolb cycle features for medical e-learning. Concrete experience: medical residents complete interactive clinical cases on the e-learning platform, reflective observation: discussion and clinical reasoning with experienced clinicians, active experimentation: application of newly acquired knowledge during clinical activity, and abstract conceptualization: further knowledge acquisition and clinical case creation. Created in BioRender [33].

This formative pilot study evaluated the utility and usability of a novel blended e-learning tool. The tool received an overall positive evaluation. Purposefully designed for users, it marks a shift from traditional, teacher-centered methods to interactive, problem-based, student-centered learning [30]. Our e-learning tool follows Kolb learning cycle (Figure 3). Medical residents complete interactive clinical cases on the platform (concrete experience), discuss and reflect with experienced clinicians (reflective observation), apply acquired knowledge during clinical activities (active experimentation), and conduct literature research to deepen their understanding or create clinical cases (abstract conceptualization). To further engage the Kolb cycle and enhance learning, learners could be invited to create e-learning content in the future (Figure 3). According to Miller pyramid, this leads to the “Performance” and “Action” levels, the end points of the learning process [2]. During e-learning sessions, residents worked asynchronously on clinical scenarios and formative assessments, receiving immediate feedback through a flipped classroom teaching method. Residents were also encouraged to discuss cases with the specialists who authored them during face-to-face learning, thereby forming a blended learning style. The observed outcomes likely reflect the combined effect of the e-learning tool and expert support; results may differ in fully self-directed settings. Our study supports adult learning theory [31] through the presentation of clinical situations to self-directed, motivated learners, as well as Kolb experiential learning model [3] and Piaget theory of cognitive development [32], fostering active experimentation and new learning rather than focusing solely on learning outcomes.

On the basis of the responses and evaluations from both questionnaires (MEES and UEQ), we identified several elements that are important to users in an e-learning environment. Aspects such as purpose, content accuracy, an appropriate level of difficulty, a sense of importance, and seriousness all contributed to increased motivation. Insisting on these aspects when presenting an e-learning tool appears crucial for motivating users. Learning enhancers, such as providing key learning messages, are crucial for boosting learning. Interactivity in e-learning was also rated highly by users. We recommend adding these enhancers when creating an e-learning tool.

Another identified strength is feedback during face-to-face interactions between learners and experienced clinicians. Vallée et al [8] recently conducted a systematic review and meta-analysis of 56 studies (N=9943) showing significantly better knowledge outcomes for blended learning (standardized mean difference 1.07, 95% CI 0.85-1.28) than for traditional learning in health education. In another study conducted during the COVID-19 pandemic, Frenck et al [34] reported that high-quality online platforms can effectively replace traditional lectures, but the development of clinical skills and professional identity requires immersive face-to-face or team-based instruction.

Finally, our content and examples were considered translatable to users’ daily work. This validates the ESPE syllabus as the basis for topic selection. Having up-to-date, accessible content sources is essential for creating applicable material that supports daily work needs.

We observed some variability in users’ responses across both questionnaires, with some users holding opposing opinions. Some users identified the inability to create their own learning path as a barrier, whereas most users selected personalization of e-learning as a learning enhancer (Textbox 1). Dependability received the lowest rating (UEQ), assessing users’ feelings of control, security, and predictability when using the e-learning platform. There was no apparent connection between negative responses on the UEQ and users’ feedback on the MEES. One explanation could be the user guide; 4 (31%) users cited a lack of navigation tools as a barrier (Textbox 1). Among these, only 1 rated expectations and security negatively, while positively assessing predictability. The user guide may have been unclear or complicated to access, contributing to feelings of insecurity and unpredictability. Comments in the MEES highlighted uncertainty about the order in which the e-learning modules should be completed, supporting this idea (comments 4 and 5 in Textbox 1). The variability in user responses to the e-learning platform may stem from differing levels of e-learning experience, which affect expectations and perceptions of novelty and creativity. Furthermore, Cronbach α values for the dependability and novelty scales were <0.7. This may reflect the small number of items per subscale and weaker interitem correlations, suggesting more heterogeneous perceptions of these constructs among participants.

Additionally, there was no clear relationship between users’ evaluations and the barriers identified in the MEES. This indicates that prior e-learning experiences and personal expectations likely shape users’ assessments. For future evaluations, it is important to consider users’ previous experiences to better understand their impact on learning outcomes and to provide clear instructions that may reduce feelings of insecurity. In our project, although a user guide was available, not all participants accessed it. To address this, we uploaded the guide to the knowledge-sharing website [35], enabling 24/7 access before use of the e-learning platform.

Addressing concerns about personal information security is also important and will help enhance trust in the platform. Our e-learning platform was designed to work on computers, tablets, and smartphones. However, it seemed computers and tablets were the most suitable devices. We do not have user data specifying the exact reasons (eg, responsive design issues). Further adapting e-learning for mobile devices could be an attractive solution. Although most users did not identify weaknesses, we recognize areas for improvement to enhance platform functionality and the user experience. These will be addressed in future projects.

Compared to other e-learning solutions, such as AMBOSS [36], which is subscription-based, our platform is freely accessible to all pediatric residents and students at Lausanne University Hospital. The United States Medical Licensing Examination e-learning platform offers practice clinical cases, but its approach is specific to that examination [37]. The ESPE also provides an e-learning platform that is freely accessed [38]. It combines theoretical knowledge and interactive clinical cases. However, it does not integrate the specificities of local clinical care. One key advantage of our solution, especially compared to AI-based approaches, is that the content is created directly by tutors based on real cases. Residents can discuss these cases in detail, enabling a more personalized approach.

### Limitations

As pediatric endocrinology is a specialized subspecialty with limited staffing, fewer residents were available to test our e-learning tool, resulting in a smaller sample size for our study. We acknowledge this as a significant limitation. This also partly explains the width of our CIs (Table 1), as these are influenced by sample size. However, our sample of users represents the entire cohort available at the time. Different challenges were encountered regarding the creation of the e-learning tool (eg, time constraints related to the development of e-learning cases and the time required for learning). These barriers, along with their potential solutions, are summarized in Multimedia Appendix 3.

### Conclusions

Our approach addresses the challenge of imparting specific knowledge that should be retained, remembered, and effectively interpreted throughout the postgraduate learning phase, while accounting for differences in learning styles and approaches in individual learning processes.

Our results support that e-learning combined with personal feedback can be an effective format for blended learning. Furthermore, our study emphasizes the importance of incorporating key messages, feedback, real-world applications (eg, clinical cases), interactivity, and customizable content into an e-learning tool. Additionally, maintaining up-to-date content is crucial for ensuring reliable information.

Further development of e-learning is encouraged, particularly for subspecialties with short residency periods, as our study has proven it to be a valuable tool. Having content developed and validated by senior specialists ensures tutor-validated material that can be discussed directly with experts, providing added educational value and supporting the development of clinical skills and professional identity. We highly recommend that learners actively contribute to the creation of e-learning tools (eg, clinical cases), as this supports sustainable e-learning environments. Evaluating user experience, motivators, and barriers is deemed essential before focusing on learning outcomes, as these factors significantly shape the learning process. Although we did not have the opportunity to include APAs in our e-learning tool, they might be another useful addition, as they have been shown to be effective [14,15]. We also encourage the use of the MEES questionnaire for further evaluation of and comparison among instructional designs, as it proved to be a valuable and practical tool for evaluating e-learning in medical education. MEES assessed Kirkpatrick level 1 (reaction) and part of level 2 (learning), focusing on motivators and barriers to measure reactions, while the UEQ evaluated attractiveness and stimulation (Kirkpatrick level 1). Although our pilot study did not directly measure acquired knowledge, the MEES examined factors that enhance or hinder the learning process. The next step would be to measure the impact on clinical practice.

Additionally, more longitudinal evaluations are needed to assess Kirkpatrick levels 3 and 4 to achieve a comprehensive understanding of the effectiveness of e-learning and blended learning, as most existing studies focus on levels 1 and 2 [39]. Although these evaluations require more resources and time, they should be prioritized by faculties and university hospitals to enhance education and ultimately improve patient care.

## Supplementary material

10.2196/89064 Multimedia Appendix 1 Overview of the e-learning platform.

10.2196/89064 Multimedia Appendix 2 Mean evaluation scores for each domain of the Medical E-learning Evaluation Survey reported by the 13 participants (12 medical residents and 1 medical student) in the formative evaluation of the pediatric endocrinology e-learning tool.

10.2196/89064 Multimedia Appendix 3 Barriers and solutions for the implementation of e-learning.

10.2196/89064 Checklist 1 iCHECK-DH checklist.

## Abbreviations

APA: animated pedagogical agent
ESPE: European Society for Paediatric Endocrinology
H5P: HTML5 package
ICAP: Interactive, Constructive, Active, and Passive
iCHECK-DH: Guidelines and Checklist for the Reporting on Digital Health Implementations
MEES: Medical E-learning Evaluation Survey
UEQ: User Experience Questionnaire
