# Supplementary material for: Development and Usability Evaluation of an E-Learning Tool for Blended Learning in Pediatric Endocrinology: Formative Pilot Study
Source: JMIR Form Res. 2026 Jul 21;10:e89064. doi: 10.2196/89064 (PMC13386660; doi:10.2196/89064)
Supplement: Multimedia Appendix 1 [file formative-v10-e89064-s001.pdf]

MULTIMEDIA APPENDIX 1 : Overview of the e-learning platform

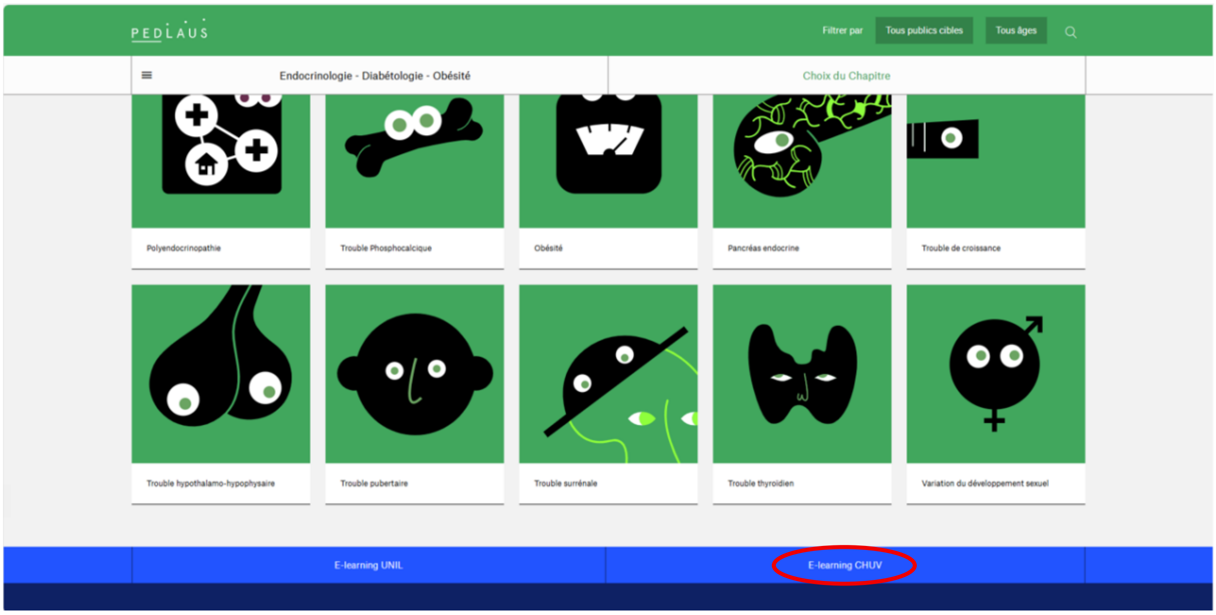

Figure S1. Freely accessible specific knowledge sharing website (computer view), including link to the e-learning website. Site and Icons designed by the Lausanne University Hospital Audiovisual communication and creation service.

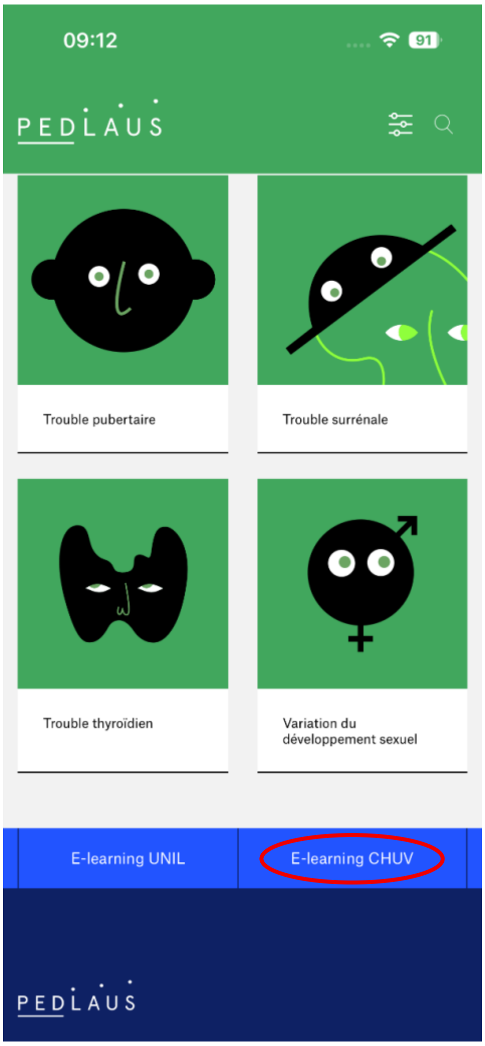

Figure S2. Specific knowledge sharing website (smartphone view) including link to the e-learning website. Site and Icons designed by the Lausanne University Hospital Audiovisual communication and creation service.

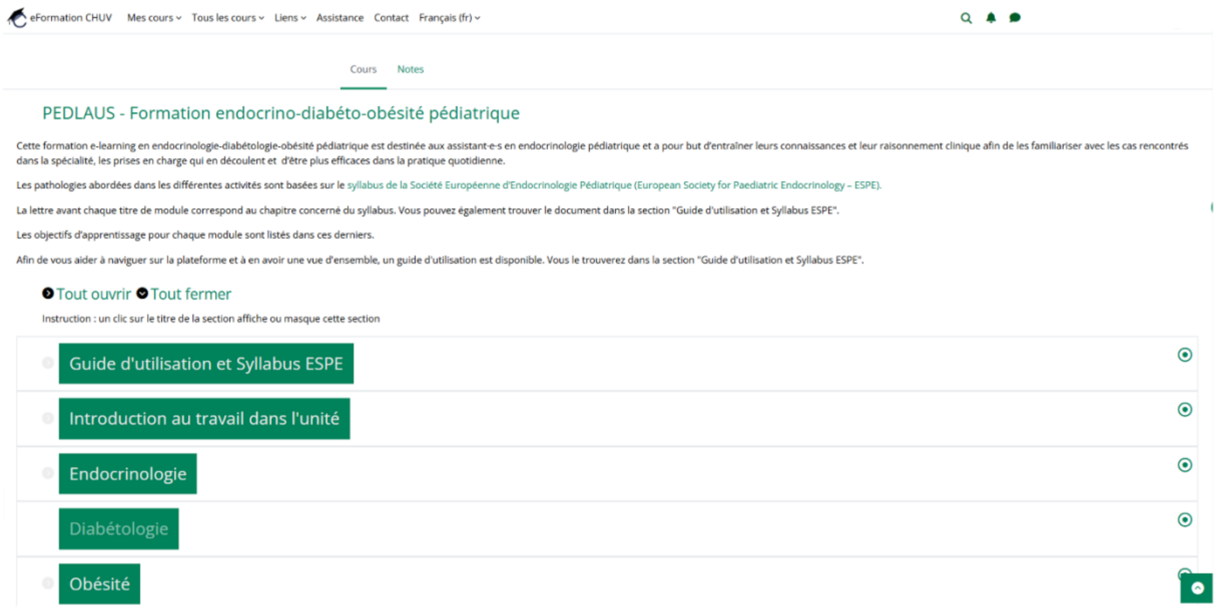

Figure S3. E-learning platform accessed directly from the knowledge sharing website to the Lausanne University Hospital e-learning platform (based on Moodle®), requiring login and personal inscription.
