# Supplementary material for: Development and Usability Evaluation of an E-Learning Tool for Blended Learning in Pediatric Endocrinology: Formative Pilot Study
Source: JMIR Form Res. 2026 Jul 21;10:e89064. doi: 10.2196/89064 (PMC13386660; doi:10.2196/89064)
Supplement: Multimedia Appendix 2 [file formative-v10-e89064-s002.pdf]

MULTIMEDIA APPENDIX 2 : Mean evaluation scores MEES

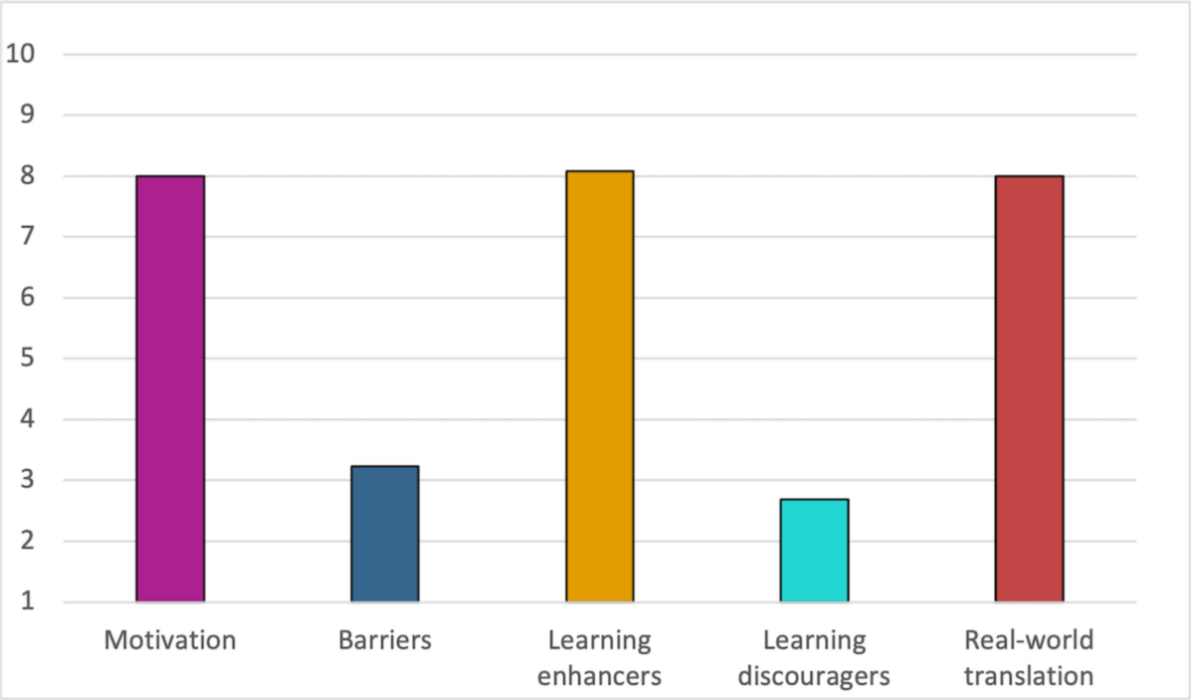

Figure S1. Mean evaluation scores for each domain of the Medical E-learning Evaluation Survey (MEES) reported by the 13 participants (12 medical residents and 1 medical student) in the formative evaluation of the pediatric endocrinology e-learning tool.
