# Supplementary material for: Development and Usability Evaluation of an E-Learning Tool for Blended Learning in Pediatric Endocrinology: Formative Pilot Study
Source: JMIR Form Res. 2026 Jul 21;10:e89064. doi: 10.2196/89064 (PMC13386660; doi:10.2196/89064)
Supplement: Multimedia Appendix 3 [file formative-v10-e89064-s003.pdf]

**Table S1. Barriers and solutions for the implementation of e-learning.**

| | Barriers | Possible Solutions |
|---|---|---|
| **Faculty/staff** | Cultural resistance | Convincing concept of technology, didactics, support. |
| | Negative attitude or reluctancy | Engagement with e-learning. |
| | Resistance to novel technology | Time to devote to the mastery.<br>Development and implementation of online learning tools. |
| | Technical skills deficit | Training skills to the faculty. |
| | | Dedicated staff / students with technical skills to facilitate the development of LCMS or wiki environment. |
| | Institutional support | Institutional Strategies.<br>Faculty reward and acknowledgement for efforts. |
| | Costs | Institutional Support.<br>Collaboration with partnering hospitals for maintenance cost.<br>Grant paid access to learners from other institutions. |
| | Time constraints | Protected administrative time.<br>Include medical students / junior staff in the concept phase.<br>Artificial intelligence to help in creation process. |
| | Poor communication | Personal contact and exchange.<br>Include relevant stakeholders and departments within a faculty to facilitate collaboration. |
| **Learner** | Digital literacy | There is a desire for more hybrid teaching. |
| | Technical skill deficit | Learners seem to have good technical skills to master LCMS. |
| | Infrastructure | Desire to personalize the e-learning platform : U bookmarks, individual calendar, annotations, guidance, joining files , support of group work via whiteboard. |
| | Time constraints | Protected learning time. |
| | Poor communication | Inclusion of Almuni in the administration, organization and editorial management process. |
