# Supplementary material for: Development and Usability Evaluation of an E-Learning Tool for Blended Learning in Pediatric Endocrinology: Formative Pilot Study
Source: JMIR Form Res. 2026 Jul 21;10:e89064. doi: 10.2196/89064 (PMC13386660; doi:10.2196/89064)
Supplement: Checklist 1 [file formative-v10-e89064-s004.pdf]

Multimedia Appendix 1. Checklist of iCHECK-DH guidelines. iCHECK-DH: Guidelines and Checklist for the Reporting on Digital Health Implementations.

SECTION		ITEM	DESCRIPTION
**TITLE**	1	Title (M[1])	"Development and usability of an e-learning tool for blended learning in pediatric endocrinology: a formative pilot study." The title describes the implementation (development of an e-learning tool for blended learning in pediatric endocrinology). The study type is identified ('formative pilot study')
**ABSTRACT**	2	Abstract (M)	Abstract is structured as suggested : background (brief residencies in subspecialties, such as pediatric endocrinology, limit exposure to diverse clinical cases; e-learning can address this), objectives (to evaluate the utility and usability of a blended learning tool), methods (blended learning model based on Kolb's cycle; case-based asynchronous e-learning modules with formative assessments; Kirkpatrick levels 1 and 2 evaluated via MEES and UEQ), results (13 users evaluated; main strengths were clinical applicability, timely summaries, and immediate feedback; main weaknesses were device compatibility and limited personalization; UEQ showed highest ratings for attractiveness and stimulation), conclusions (tool is effective and motivating for postgraduate medical education in pediatric endocrinology; longitudinal evaluation still needed).<br>Keywords : medical education; e-learning; blended learning; andragogy; implementation
**INTRODUCTION**	3	Context (M)	The introduction provides with the context of the development of the -learning tool for blended learning in pediatric endocrinology as a subspecialty at the Lausanne University Hospital. The medical residents from the pediatric endocrinology are the target group.<br><br>Definition of e-learning. E-learning described as a way to offer complementary opportunity for asynchronous blended learning. Introduction of an e-learning tool with interactive case-based learning.
	4	Problem statement (M)	Challenge in medical postgraduate education, especially during short-term residencies such as pediatric endocrinology, is to experience enough clinical situations in order to acquire evidence-based practical knowledge, clinical skills, and medical attitude, which is essential in a medical professional's journey.
	5	Similar Interventions (M)	It is a pilot study as described in the introduction.
**METHODS**	6	Aims and Objectives (M)	Our e-learning tool was evaluated using the Postgraduate Evaluation Survey for Medical E-learning (MEES) and the User Experience Questionnaire (UEQ). These instruments enabled us to assess Kirkpatrick's level 1 and part of level 2. As we completed a formative assessment of our e-learning tool, we did not evaluate achievement of the learning objectives. We evaluated our e-learning tool in a blended learning environment, combining asynchronous self-directed e-learning with optional face-to-face discussions with senior specialists.
	7	Blueprint summary (M)	The e-learning tool is implemented on the Lausanne University Hospital e-learning platform. Chapter structure, the selection of topics addressed in the e-learning, and the formulation of specific learning objectives are based on the syllabus in Pediatric Endocrinology, published by the European Society for Pediatric Endocrinology (ESPE). For the clinical cases, data from real patient whose consent and/or parental consent for data collection had been obtained previously at the Lausanne University Hospital.
	8	Technical Design (M)	As the Lausanne University Hospital already has an a-learning platform (Moodle®), we used it to implement the pediatric endocrinology e-learning platform. We used the open source H5P (HTML5 Package) module integrated into the Moodle Platform

[1] M: Mandatory item

			to transform clinical situations into structured interactive content. The e-learning tool is linked to a specific content sharing openly accessible website.
	9	Target<br>(M)	The target are the medical residents from the pediatric endocrinology unit at the Lausanne University Hospital.
	10	Data<br>(M)	For the clinical cases data from real patients was used for whom consent and/or parental consent for data collection had been obtained previously at the Lausanne University Hospital.<br><br>To access the e-learning platform login and personal registration are needed.Describe the data governance, including life cycle (collection, processing, storage, modification, sharing, suppression), the data ownership (mention whether patients actually have access to the data), data protection measures, confidential use of routine data, expected level of data integration, data for research, cross-border data agreement, if any, the applicable legal framework, and how the project complies with it. Data consent: Has patient consent been obtained? Describe the approach to data protection and cybersecurity (e.g. security by design, privacy by design, etc.) and where the data is hosted. (e.g., in-country, cloud based, hybrid model etc.). Describe, if applicable, the government preferences in terms of data policies.
	11	Interoperability<br>(M)	Open-source e-learning platform (Moodle®), and open source H5P (HTML5 Package) module integrated into the Moodle Platform were used
	12	Participating entities<br>(M)	Implementing organization(s): Pediatric endocrinology, diabetology and obesity unit, Service of Pediatrics, Lausanne University Hospital, Lausanne Switzerland<br><br>Government involvement: no direct involvement from the government.<br><br>Partners: Describe all partners (organisations) and their role in the implementation.<br><br>Funders: no specific funding for this implementation..<br><br>Which entity will own the final product and intellectual property after the implementation phase : Lausanne University hospital
	13	Budget Planning<br>(M)	The e-learning platform is hosted and supported by the Lausanne University hospital. No additional costs are expected.
	14	Sustainability<br>(M)	The e-learning platform is hosted and supported by the Lausanne University hospital. No additional costs are expected.
**RESULTS**	15	Coverage<br>(M)	The coverage is limited to the medical residents from the pediatric endocrinology unit at Lausanne University Hospital, and to 1 medical student.
	16	Outcomes<br>(M)	For the e-learning tool : 27 interactive clinical cases based on real patients. 13 participants tested and evaluated the tool with the 2 questionnaires (MEES and UEQ).<br>Questionnaires : Evaluations show a globally positive evaluation. Results from each questionnaire are described in the section.
	17	Lessons learned<br>(M)	Describe any lessons learned from the implementation experience that could be used to improve future outcomes. This could include, but is not limited to, success factors, implementation challenges or budget considerations.

			Success factors: involvement of medical senior staff/senior teachers, opportunity to discuss face-to-face with the senior teachers about the cases. Interest and motivation from medical residents for e-learning.<br><br>Challenges to implementation: especially time-constraints making the process of collecting clinical cases longer<br><br>What recommendations can be drawn from the lessons learned ? Time constraints, protected learning and administrative time. Institutional support.
	18	Unintended consequences (NM[2])	None.
**DISCUSSION**	19	Conclusion (M)	Conclusions and future implications are summed up at the end of the discussion. Main conclusions : our approach tackles the challenge of short-term medical residencies with fewer time to see various clinical situations. E-learning is an appreciated format and is effective for blended learning. Next step would be to measure the impact on clinical practice. Further development of e-learning is encouraged, important to have longitudinal studies that can achieve a comprehensive understanding of e-learning's effectiveness.
**GENERAL**	20	General (NM)	Not applicable

[2] NM : Non-mandatory item
